# Effect of Polymer Blends on the Properties of Foamed Wood-Polymer Composites

**DOI:** 10.3390/ma12121971

**Published:** 2019-06-19

**Authors:** Suwei Wang, Ping Xue, Mingyin Jia, Jing Tian, Run Zhang

**Affiliations:** College of Mechanical and Electrical Engineering, Beijing University of Chemical Technology, Beijing 100029, China; wangsw90@163.com (S.W.); jiamy@mail.buct.edu.cn (M.J.); tianjing_buct@163.com (J.T.); zhangrun_buct@163.com (R.Z.)

**Keywords:** wood-polymer composites, polypropylene, extrusion foaming, polymer blends, chemical blowing agent

## Abstract

The polypropylene (PP)/wood flour (WF) composites were prepared using a co-rotating twin-screw extruder followed by a single-screw extruder foaming system in this paper. Polymers, such as polyolefin elastomer (POE), high-density polyethylene (HDPE) or microcrystalline wax, were blended with PP in the preparation of composites to improve the melt strength. And a cavity transfer mixer was introduced to increase the distribution uniformity of components in composites. Meanwhile, the effect of the polymer blends on the microstructure and mechanical properties of samples was investigated. The experimental results show that the addition of POE and HDPE resulted in the second melting peak in the differential scanning calorimeter (DSC) curves and a great decrease in the cell size was caused by the added POE. However, due to the velocity difference of composites in the die, the shape of bubbles gradually became irregular. Moreover, the impact strength of samples significantly increased by 85% for the added POE and the apparent density decreased by 6.7%. And the minimum Vicat softening temperature of 133.7 °C was obtained when the mass ratio of HDPE to PP was 4/6.

## 1. Introduction

Compared with neat polymers, the wood-polymer composites have advantages in material costs and stiffness, which helps wood-polymer composites to be gradually accepted by the plastics industry [1]. However, these advantages are obtained at the expense of a loss in ductility and impact strength of the composites, and the addition of wood flour will lead to a higher density of composites compared to neat polymers and wood, which have limited the use of wood-polymer composites in some areas in recent years.

Therefore, a uniform microcellular structure was introduced into the wood-polymer composites to improve ductility and decrease density. This can be attributed to the fact that small bubbles can effectively suppress crack growth by passivating the crack tip and increasing the energy required for crack growth. As a result, the products with microcellular structure can be used in automotive, high-speed rail, aerospace, military and other fields.

However, most of the wood-plastic foamed products on the market use amorphous polymer as the resin matrix, such as polyvinyl chloride (PVC) [2,3], polystyrene (PS) [4]. This is because the melt strength of amorphous polymers decreases slowly with the increase of processing temperature during the transition from high elastic state to viscous flow state, which makes PVC and PS can effectively restrain the excessive expansion of bubbles in the foaming process. Compared with the amorphous polymers, the melt strength of crystalline polymers decreases rapidly after the processing temperature exceeds the melting temperature, which leads to a narrow temperature range for the melt strength of crystalline polymers to meet the foaming requirements. The temperature range of PP is just 4 °C, while the temperature range of PS is 50 °C. In the extrusion process, the actual temperature of the material is difficult to control accurately. And the narrow temperature range will make it difficult for the melt of PP to support the excessive expansion of the bubbles in the foaming process, which will cause bubble collapse, bubble coalescence and other defects. So PP is seldom used to prepare microporous plastics, although it has lower density, higher water and chemical resistance, better mechanical properties and higher operating temperature [5,6,7].

In order to achieve a uniform microcellular structure, many researches were focused on blending PP with other polymers to improve the melt strength, such as HDPE, polybutylene terephthalate (PBT), polytetrafluoroethylene (PTFE) and so on. The introduction of HDPE would increase the solubility of CO_2_ and decrease the crystallinity of both HDPE and PP, which contributed to facilitate the production of microcellular foam in blend materials [8]. Besides, the addition of PBT acts as heterogeneous nucleation during crystallization and the physical network formed by the entanglement of fibrous PBT can effectively restrain the growth of bubbles in the foaming process, which helps to reduce cell size as well as improve cell density and cell uniformity [9]. Compared with HDPE and PBT, the fibrous PTFE can not only enhance the CO_2_ sorption capacity of the matrix, but also form a physical network in the melt to restrain the bubbles, which resulted in a great increase in bubble density and volume expansion ratio [10,11]. The samples with microcellular structure mentioned above are prepared by batch foaming process. In contrast, the cell size and cell density of samples prepared by microcellular continuous extrusion are at least one order of magnitude different from those of samples prepared by batch foaming. In addition, most of the studies concerning microcellular continuous extrusion of PP are based on capillary die with a diameter less than 2 mm, and there are few studies that focus on microcellular continuous extrusion of PP/WF composites.

Therefore, on the basis of previous studies, PP/WF composites will be prepared by microcellular continuous extrusion in this article based on the die with large rectangular section. And in the previous studies, POE was often used to improve the ductility of composites, although the effect of POE on the cell morphology was seldom investigated [12,13]. Besides, in the previous experiments, the fact that the addition of a small amount of microcrystalline wax helped to obtain the smaller bubbles was found, which was also seldom investigated.

Moreover, in order to improve the cellular structure, it is necessary to increase the distribution uniformity of materials to obtain the polymer/gas homogenous system, which mainly depended on the introduction of static mixer and the enhancement of screw shear action in the previous research [13,14]. However, the addition of wood flour would increase the viscosity of composites and made the mixing of composites more difficult. Besides, the previous work indicted that the static mixer used in the preparation of wood-plastic composites was easy to deform for the high extrusion pressure. As a result, the cavity transfer mixer (CTM) [14] was introduced to replace the role of static mixer in the preparation of wood-plastic composites. What’s more, the melt pump was also introduced to maintain the stability of extrusion pressure.

In this study, the blends of PP with POE, HDPE and microcrystalline wax were prepared respectively and selected as a polymeric matrix. And a cavity transfer mixer was mounted to achieve a single-phase polymer solution with higher distribution uniformity in the foaming process. Then, the effect of polymer blends on the thermal and crystalline performance and rheological property of the composites was investigated. Besides, the effect of polymer blends on the cell morphology and mechanical properties of samples was also investigated.

## 2. Experimental

### 2.1. Materials

The samples studied in this article were mainly prepared from materials such as polymer blends, wood flour (WF), inorganic filler and blowing agent. The polymer blends were made by blending PP with POE, HDPE and microcrystalline wax, respectively. PP, B1002W with the density of 0.902 g/cm^3^ and the melt flow index of 1.0 g/10 min at 230 °C/2.16 kg, was obtained from SINOPEC (Beijing, China). HDPE, 5000S with the density of 0.950 g/cm^3^ and the melt flow index of 0.9 g/10 min at 190 °C/2.16 kg, was obtained from SINOPEC, China. POE, DF605 with the density of 0.862 g/cm^3^ and the melt flow index of 0.5 g/10 min at 190 °C/2.16 kg, was obtained from Mitsui Chemicals (Tokyo, Japan). Microcrystalline wax, 155 with the density of 0.783 g/cm^3^ and the melting temperature of 69.3 °C, was obtained from Nippon Seiro Co. (Tokyo, Japan). Besides, the poplar wood flour with the grain size of 150 μm (100 mesh) was obtained from HC wooden Co. (Jiangshan, China). And the nucleating agent, talc was obtained from Haicheng Talc Powder Manufacturer, China, with the grain size of 23 μm (600 mesh). In order to improve the interface between polymer blends and wood flour, maleic anhydride-g-PP (MAH-g-PP, D201) used as the coupling agent was obtained from Shanghai Bingo International Trading Co. (Shanghai, China), with the melt flow index of 150 g/10 min at 230 °C/2.16 kg. At last, the blowing agent, Azodicarbonamide (AC) was obtained from Selon Industrial Co. (Leping, China), with the gas production of 220 mL/g and the decomposition temperature of 160 °C.

### 2.2. Composites Preparation

First, in order to improve the melt strength of the polymer matrix, PP was blended with other polymers in a high-speed mixer (SHR-25A, Zhangjiagang Yongli Machinery Co., Ltd., Suzhou, China) for 4 min, such as HDPE, POE and microcrystalline wax. Then, the mixtures were melt-extruded using a 20-mm-diameter co-rotating twin-screw extruder with the length/diameter ratio of 40 (Kunshan Kesun, Rubber & Plastic Machinery Co., Kunshan, China). At last, the molten polymer was cooled by air and then fed into a pelletizer to obtain the resin pellets with uniform size.

Second, the wood flour dried at 80 °C for 24 h was blended with resin pellets, talc and MAH-g-PP in a high-speed mixer for 30 min. The mass ratio of polymer blends, wood flour, talc and MAH-g-PP was 70:30:10:3.5 in this study. Besides, the compositions of polymer blends for different samples are listed in Table 1. Then, similar with the steps above, the mixture was melt-extruded using a co-rotating twin-screw extruder to further improve the uniformity of distribution of wood flour in composition. And the WPC pellets with uniform size were obtained after the extrudate was fed into a pelletizer.

During the extrusion process, the zone temperatures and rotational speed of the twin-screw extruder were set at 130, 150, 170, 185, and 170 °C (from hopper to die) and 150 rpm, respectively.

### 2.3. Extrusion Foaming

**Figure 1:**
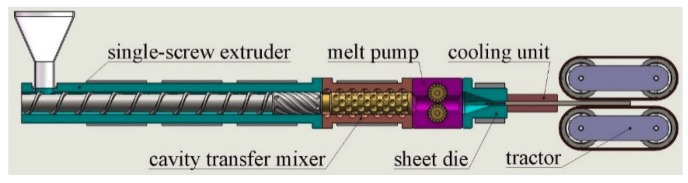
Schematic diagram of the single-screw extruder foaming system.

In this study, the foaming samples were prepared by a single-screw extruder foaming system, which was shown in Figure 1. The foaming system mainly consisted of a 45-mm-diameter single-screw extruder with the length/diameter ratio of 25 (Shanghai Sanlei Plastic Machinery Manufacturing Co., Ltd., Shanghai, China), a cavity transfer mixer, a melt pump (ZB-B3-20CC, Batte Melt Pump Zhengzhou Co., Ltd., Zhengzhou, China), a sheet die with a rectangular outlet section and a tractor. The rectangular outlet section of die was 80 mm in width by 8 mm in height, respectively.

Before extrusion, the WPC pellets were blended with the AC foaming agent at a mass ratio of 100:0.5 in a high-speed mixer for 3 min. Besides, the liquid paraffin was added before mixing and the powder of AC foaming agent was uniformly wrapped on the surface of WPC particles in the mixing process because of the adhesion of liquid paraffin. Then the mixture was put in the hopper to achieve a single-phase polymer solution under the strong shear action of screw mixing and the cavity transfer mixer. At last, the single-phase solution was delivered into the sheet die where the gas solubility in the polymer would decrease suddenly for the rapid pressure drop of the melt. As a result of the decrease of the gas solubility, a lot of bubbles would nucleate and growth in the resin matrix to create a foam structure. Considering the decomposition temperature of the blowing agent and the melting temperature of the WPC pellets, the temperatures of the sheet die and the melt pump were kept at 180 °C, and the zone temperatures of the extruder barrel were kept at 140, 160 and 175 °C, respectively.

### 2.4. Characterization

#### 2.4.1. Thermal Analysis

**Figure 2:**
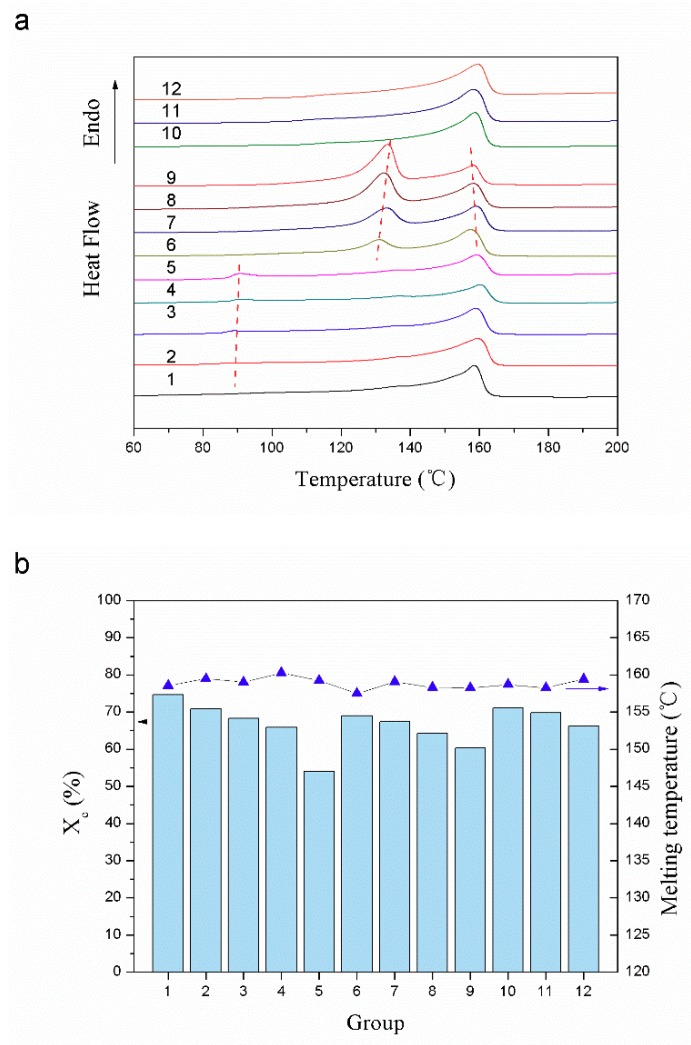
DSC thermograms and results of the samples with different polymer blends: (**a**) DSC thermograms, (**b**) DSC results.

The crystallization behavior of the samples was analyzed by a differential scanning calorimeter (Q2000, TA Instruments Co., New Castle, DE, USA). The measurements were performed in nitrogen atmosphere as following procedures: samples were heated up to 210 °C at rate of 10 °C/min and held in a molten state for 1 min to erase any previous thermal history, followed by cooling down to the room temperature at the same rate. Data for heating and cooling cycle were recorded, and then, the melting temperature (Tm) and crystallization temperature (Tc) values for the samples were read from the graphs, as listed in Figure 2. The crystallinity percentage (*Xc*) of the PP phase was calculated according to the following equation:

(1) $$X_{c}=\frac{∆H_{f}}{∆H_{f}^{o}\times \omega }$$

where $∆H_{f}$ is the heat of fusion generated by the cold crystallization, $∆H_{f}^{o}$ is the theoretical heat of fusion of 100% crystalline PP with a value of 209 J/g [9], and ω is the weight fraction of PP in the sample.

#### 2.4.2. Rheological Property Testing

The rheological properties of samples in this study were characterized by the frequency dependences of molten-state oscillatory shear moduli, such as the shear storage modulus G′ and the loss modulus G″, which were conducted using a parallel-plate rheometer (HAAKE MARS III, Thermo Fisher Scientific Inc., Waltham, MA, USA). The tests were performed in dynamic mode between parallel plates (diameter 25 mm, gap 1 mm) at 180 °C in nitrogen atmosphere. Besides, in the process of testing, frequency sweeps between 0.01 and 100 rad/s were carried out at strains within the linear viscoelastic range and the scan amplitude was 1%.

#### 2.4.3. Morphological Analysis

The microstructures of the samples were observed by a scanning electron microscope (Model S-4700, Japan Hitachi Company, Tokyo, Japan). In order to avoid damaging the surface morphology, each sample was fractured after cooling in liquid nitrogen for about 10 min and sputter-coated with gold before observation. Then the SEM photographs were analyzed by image analysis software (Image Pro Plus 6.0) to obtain the average cell size and cell density of the samples.

The number average diameter of all cells in one micrograph, $\bar{d}$, was calculated as follows:

(2) $$\bar{d}=\frac{\sum_{i=1}^{n}d_{i}}{n}$$

where *d_i_* is the diameter of a single cell, and n is the number of counted cells. At least 100 cells were selected randomly from the SEM graph of each sample to evaluate the average diameter.

The cell density *N*_0_, defined as the number of cells per unit volume of the polymer, was calculated as follows:

(3) $$N_{0}={\left[\frac{nM^{2}}{A}\right]}^{3/2}\frac{\rho_{0}}{\rho_{f}}$$

where *A* is the area of an SEM graph (cm^2^), *n* is the number of cells in the micrograph, *M* is the magnification factor, $\rho_{0}$ is the density of the unfoamed sample and $\rho_{f}$ is the density of the foamed sample [15].

#### 2.4.4. Mechanical Property Testing

All the mechanical properties of samples were tested according to standard testing methods. Both tensile tests and flexural tests were conducted on a universal tester (KXWW, Chengde Taiding Testing Machine Manufacturing Co., Ltd., Chengde, China) with a load cell of 5 kN. The tensile strengths of the samples were measured according to GB/T 1040.2-2006 with a draw speed of 10 mm/min. Meanwhile, the three-point flexural tests were conducted according to GB/T 9341-2008 with a cross-head speed of 10 mm/min [16].

Moreover, notched impact strength tests were conducted on an impact tester (KBANM-II, Chengde Taiding Testing Machine Manufacturing Co., Ltd., China) according to GB/T 1843-2008. Before testing, a side-edge notch with a depth of 2 mm was machined on each specimen. For each data point, at least 5 to 10 samples were tested in this study [17].

#### 2.4.5. Density Measurements

To measure the apparent density of each sample, the weight and volume of the sample were separately determined using an electronic balance with an accuracy of 0.01 g and a micrometer caliper with an accuracy of 0.01 mm. The weight of the sample was divided by the sample’s volume to obtain the apparent density. Besides, five replicates were used for each sample and the mean value was taken. 

#### 2.4.6. Vicat Softening Point Testing

The Vicat softening point tests were conducted on a HDT-Vicat test equipment (KXRW-300CL-3, Chengde Taiding Testing Machine Manufacturing Co., Ltd., China) with a heating rate of 50 °C/h, according to GB/T 1633-2000.

## 3. Results and Discussions

### 3.1. Thermal Property

Previous studies have shown that blending with HDPE will simultaneously reduce the crystallinity of both HDPE and PP [8,18]. As shown in Figure 2, the samples containing HDPE had two well-separated melting peaks in the DSC curves, which reflected the two crystalline phases in the composites. Besides, the peak value of PP in the DSC curves decreased with the increasing content of HDPE for that the crystalline phase of HDPE dispersed in the PP matrix would greatly destroy the regularity of the molecular chain around it [19]. Meanwhile, with the increase of HDPE content, the two well-separated melting peaks representing the melting points of PP and HDPE in the polymer blends would get close to each other, which contributed to broaden the melting range of composites.

There were also two melting peaks in the curves of samples blended with POE. But the melting peak of the POE crystalline phase was not obvious in the DSC curves, because the added POE was an amorphous polymer with low crystallinity. Besides, the added POE played the role of heterogeneous nucleation during the crystallization process, which increased the crystallization rate of PP. But the long molecular chains of POE also hindered the aggregation and rearrangement of PP macromolecular chains, which resulted in the crystal defects and reduced the crystallinity of PP [20]. According to the data in Figure 2b, the addition of POE caused a greater decrease in the crystallinity of PP at the POE content of 40% compared to HDPE.

Moreover, the addition of microcrystalline wax also caused the decrease of the crystallinity of PP. But due to the small content of microcrystalline wax, the added microcrystalline wax had a less influence on the crystallinity and the second melting peak did not appear in the DSC curves [21].

### 3.2. Rheological Property

**Figure 3:**
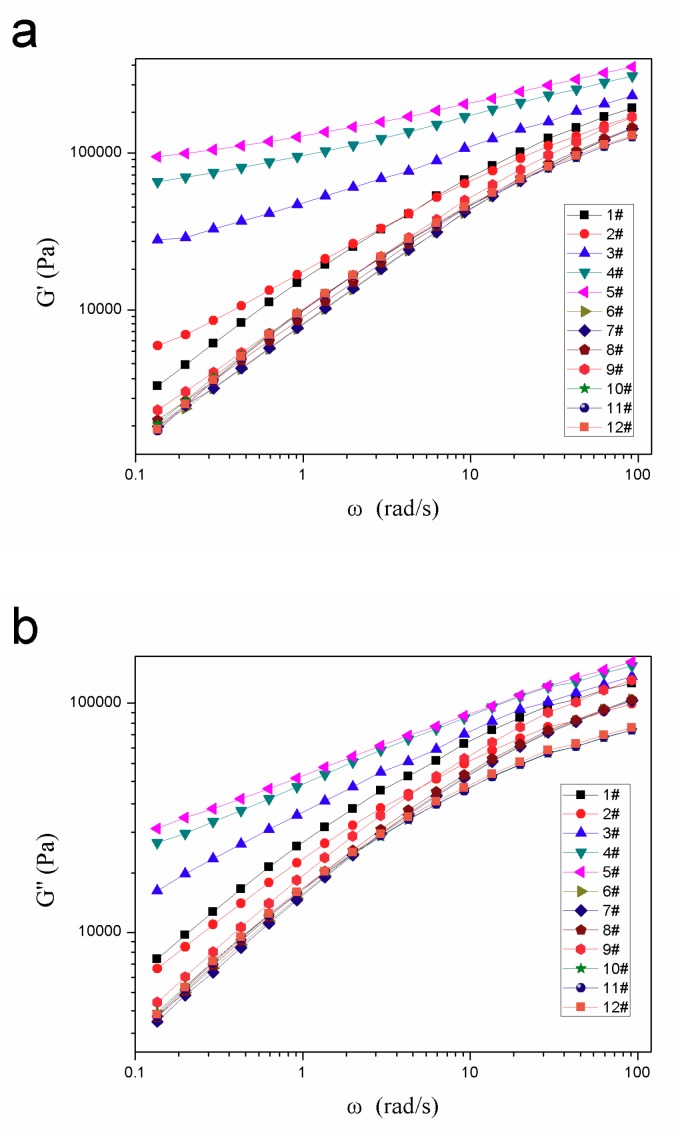

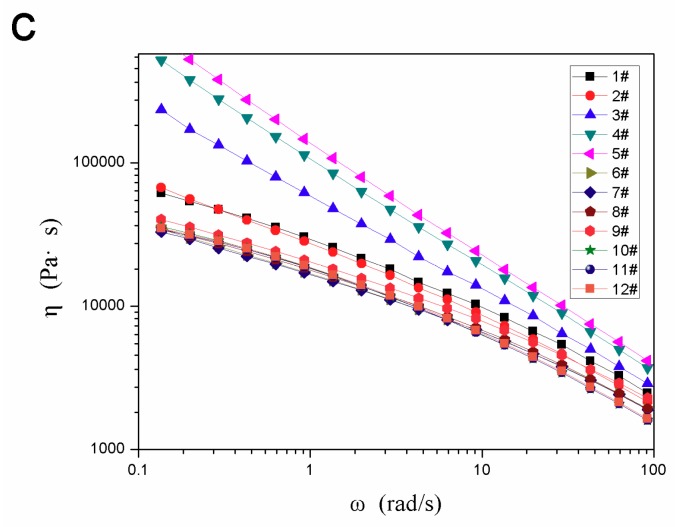
Rheological properties of the samples with different polymer blends: (**a**) storage modulus (G′), (**b**) loss modulus (G″), (**c**) complex viscosity (η).

As shown in Figure 3, the polymer blends in the composites had a significant effect on the rheological properties of samples, such as storage modulus (G′), loss modulus (G″) and complex viscosity (η). According to the data in Figure 3, the addition of POE distinctly increased both the oscillating shear modulus and the complex viscosity, especially at low frequency region. This might be attributed to the fact that the addition of POE with numerous branched chains could form a large number of physical cross-linking points with PP in the process of melt mixing, which contributed to the improvement of the melt strength of composites. Besides, with the increase of POE content, the added POE gradually became a continuous phase in the composites and the viscosity of composites also tended to be stable. When the blending ratio of POE and PP was 4/6, the shear modulus and complex viscosity of composites changed little compared with that of the sample with mass ratio of 3/7.

Compared with POE, the addition of HDPE and microcrystalline wax would melt first during the mixing process and then penetrate into PP molecules to reduce friction between molecular chains, which made the molecular chains easier to slip and rotate at the time of deformation. As a result, the storage modulus (G′), loss modulus (G″) and complex viscosity (η) of samples blended with HDPE and microcrystalline wax decreased for the lubrication of HDPE and microcrystalline wax [21,22].

### 3.3. Cell Morphology

**Figure 4:**
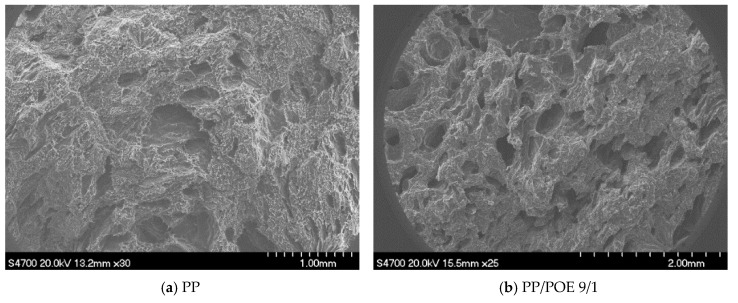

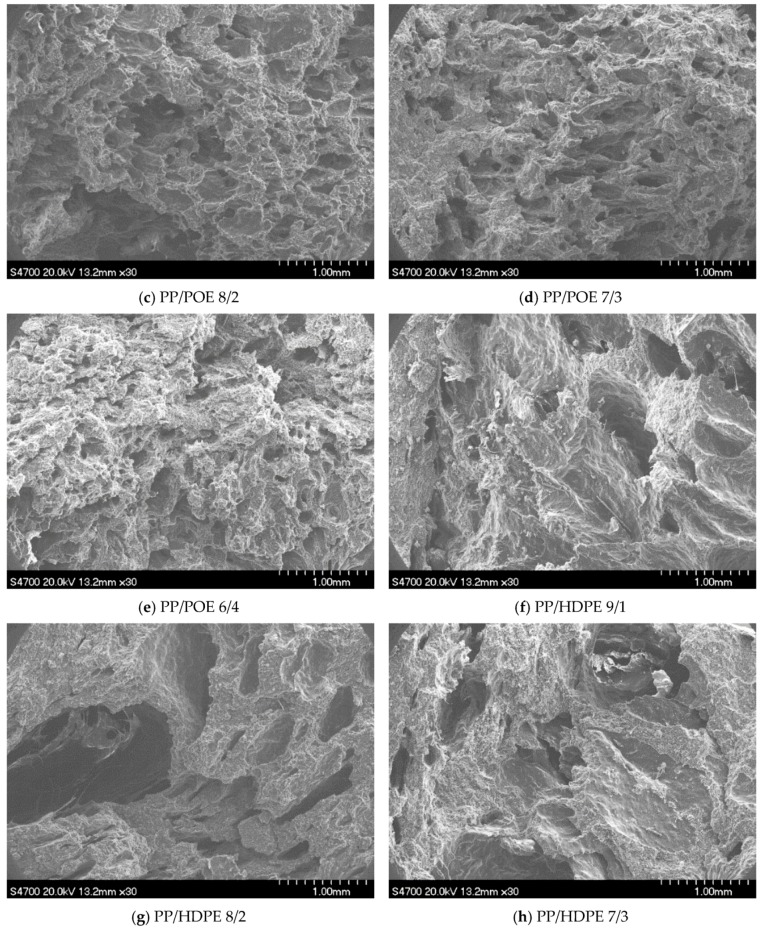

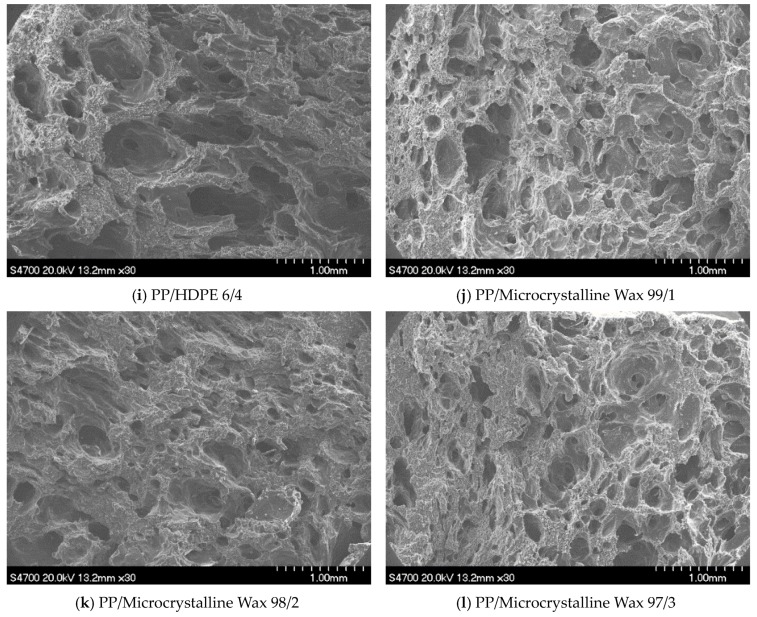
Cell morphology of the foaming samples with different polymer blends: (**a**) sample 1, (**b**) sample 2, (**c**) sample 3, (**d**) sample 4, (**e**) sample 5, (**f**) sample 6, (**g**) sample 7, (**h**) sample 8, (**i**) sample 9, (**j**) sample 10, (**k**) sample 11, (**l**) sample 12.

**Figure 5:**
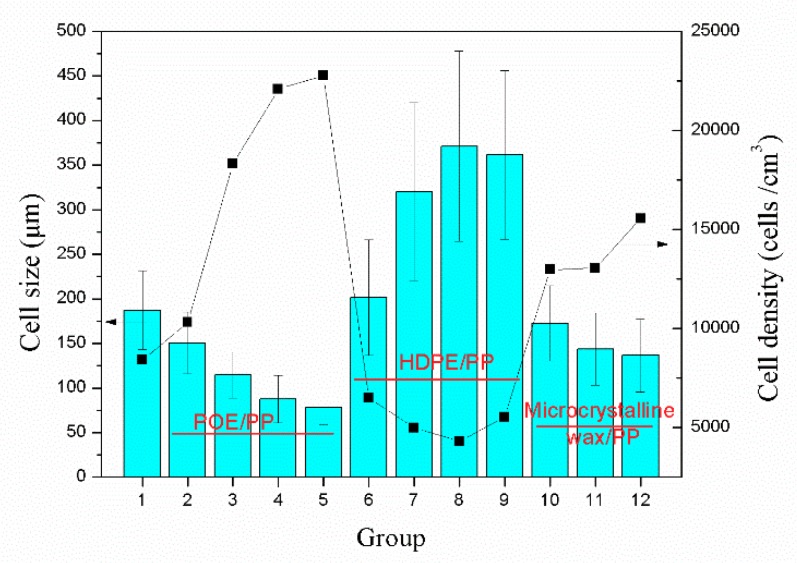
Cell size and cell density of the foaming samples with different polymer blends.

Figure 4 shows the microstructure of different samples and the cell size calculated by the micrographs is presented in Figure 5. According to the data in Figure 5, the addition of both POE and microcrystalline wax increased the cell population density and decreased the cell size of the samples. During the foaming process, the added microcrystalline wax acted as a lubricant to increase the uniformity of distribution of the wood flour in the resin matrix, which helped to avoid the combination of cells caused by wood powder agglomeration and helped to slightly improve the cell structure of the samples. Besides, the addition of POE could greatly improve the melt strength of composites, which contributed to increase the resistance of bubble expansion and avoid excessive expansion of the bubbles. When the blending ratio of POE and PP was 4/6, the cell size of sample reduced to 78 μm, which was reduced by 58% compared to the sample without blending. And the cell population density increased to 2.3 × 10^5^ cells/cm^3^, which was 170% higher than that of the sample without blending.

However, the addition of HDPE was responsible for the sharp decline in the cell population density and the great increase in the cell size. This is mainly because the reduced melt strength of the composites blended with HDPE made the cell wall become thinner during the extrusion process. On one hand, the thinner cell wall was unable to support the excessive expansion of the bubbles, which caused the merging of adjacent cells to form larger cells. On the other hand, the thinner cell wall could lead to the escape of gas used for bubble nucleation and bubble growth, which caused the collapse of bubbles and a slight decrease in the cell size [23].

According to the previous studies, the shape of bubbles in the cellular plastic prepared by batch foaming [24], injection molding foaming [25,26] or compression molding foaming is nearly spherical. However, compared with other methods, the shape of bubbles prepared by extrusion foaming was not very regular, which was mainly attributed to the fact that the materials were in a flowing state during the foaming process [27].

**Figure 6:**
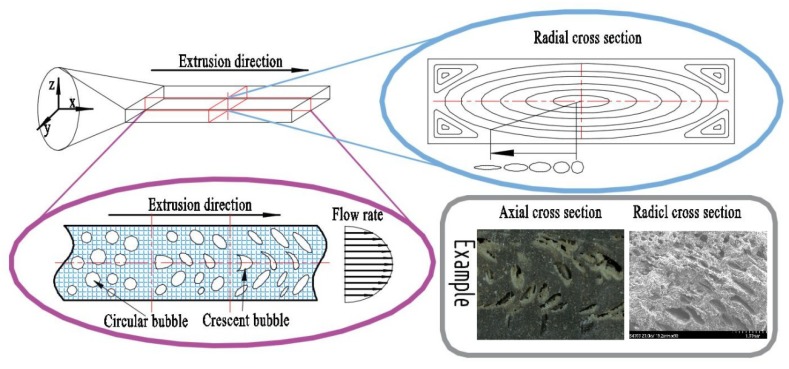
Schematic illustration of cell formation in the sheet die.

As shown in Figure 6, the molten materials in the sheet die flowed along the axial direction (x axis) under the action of the melt pump. Meanwhile, the flow rate of the materials showed parabola variation with the z axis and the maximum flow rate was achieved in the middle of flow channel. Due to the velocity difference along the z axis, the shape of bubbles on the cross section perpendicular to the y axis gradually changed from spherical to crescent during extrusion. Moreover, Figure 6 also shows the flow pattern of materials at the radial section. In the flow process, the bubbles in the flow field were gradually stretched along the flow direction into fusiform.

### 3.4. Mechanical Properties

**Figure 7:**
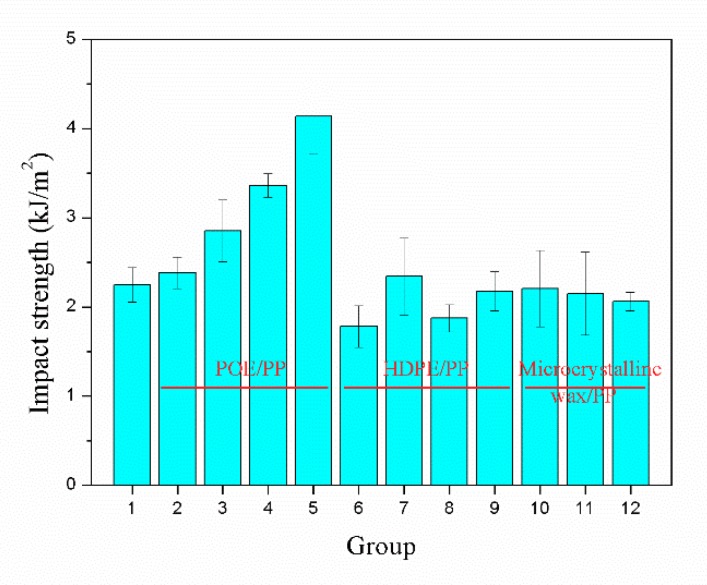
Notched impact strength of the samples with different polymer blends.

As shown in Figure 7, compared with HDPE and microcrystalline wax, the impact strength of the samples was significantly improved with the addition of POE. When the blending ratio of POE and PP was 4/6, the impact strength of the sample was 4.1 kJ/m^2^, which was 84% higher than that of the sample without blending. On one hand, the added POE helped to improve the ductility of PP/POE blend [12]. The POE particles dispersed in composites acted as stress concentration points when subjected to external impact loading, which could generate crazes and shear bands in matrix and consume a lot of energy. Meanwhile, the growth of crazes were hindered by the continuous branching of crazes and the interference of stress fields between crazes and shear bands [28]. On the other hand, the addition of POE contributed to improve the melt strength of composites, which helped to achieve a uniform microcellular structure. The microcellular structure in the samples could passivate the crack tip and thereby increase the impact strength of the samples.

**Figure 8:**
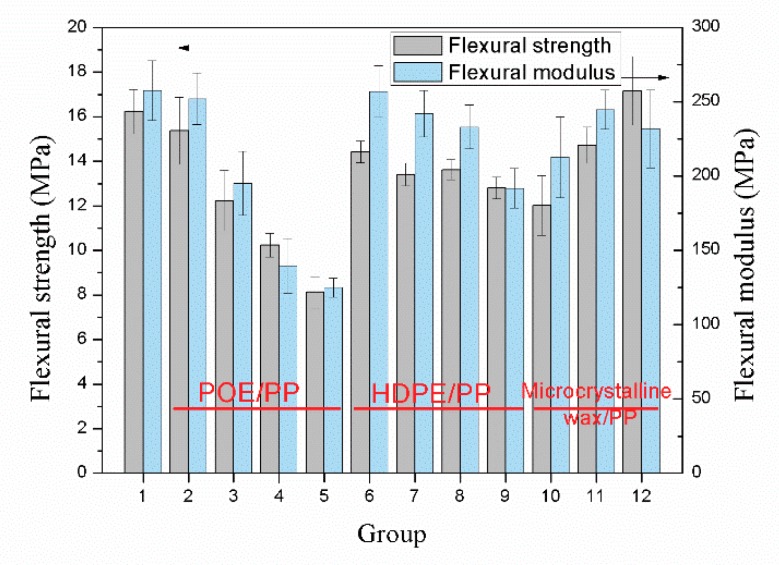
Flexural strength and modulus of the samples with different polymer blends.

**Figure 9:**
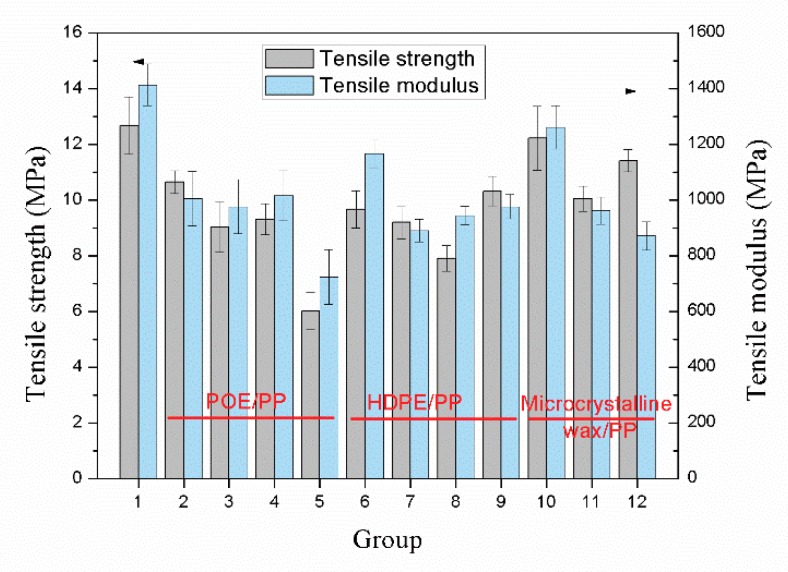
Tensile strength and modulus of the samples with different polymer blends.

As shown in Figure 8 and Figure 9, blending with POE and HDPE significantly reduced the flexural strength and tensile strength of the samples. Compared with HDPE, the addition of POE had a greater impact on the mechanical properties of the samples. When the blending ratio of POE and PP was 4/6, the tensile strength and flexural strength of the sample were 6 MPa and 8.1 MPa, respectively, which were reduced by 34% and 50%, respectively, compared to the sample without blending. This is mainly determined by the performance of the POE itself. Although the addition of POE can effectively improve the cell structure of the sample, the poor mechanical strength of the POE itself greatly weakens the tensile and bending strength of the sample [29,30]. However, the effect of HDPE is completely opposite to that of POE. Although blending with HDPE cannot improve the cell structure of the sample, the better mechanical properties of HDPE compared to POE make the tensile and bending strength of the sample decrease less. In contrast, the addition of microcrystalline wax helped to improve the mechanical properties of the samples. When the blending ratio of microcrystalline wax and PP was 3/97, the tensile and flexural strengths of the sample were 11.4 MPa and 17.2 MPa, respectively, which were 25% and 6% higher than that of the sample without blending, respectively. On one hand, the content of microcrystalline wax is small, which makes the mechanical strength of the samples less affected by the performance of the microcrystalline wax itself. On the other hand, the addition of microcrystalline wax can slightly improve the cell structure of the samples.

Therefore, in the actual production process, the determination of POE content needs to consider the effect of POE on the ductility and mechanical strength of the samples. 

### 3.5. Apparent Density and Vicat Softening Temperature

**Figure 10:**
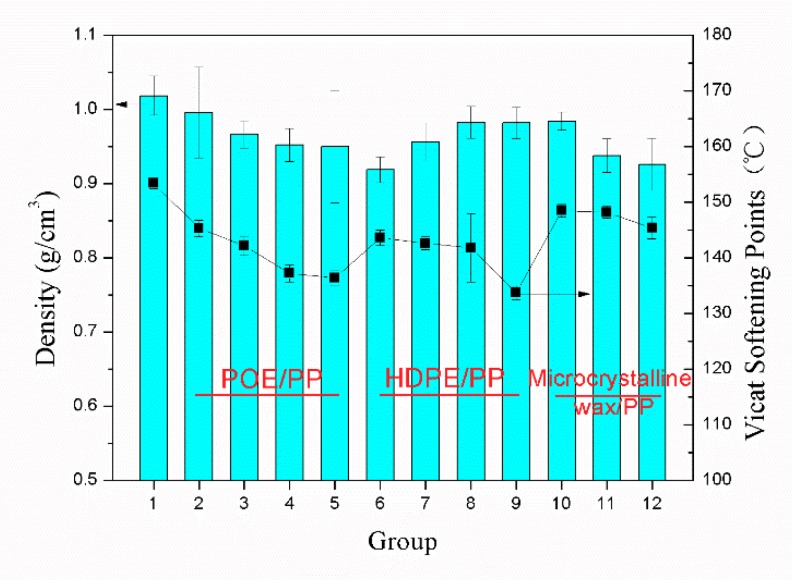
Density and Vicat softening point of the samples with different polymer blends.

Figure 10 shows the apparent densities and Vicat softening temperatures of different samples, which were affected by the compositions of the polymer blends and the microstructure of samples. 

As shown in Figure 10, the addition of POE caused the decrease of apparent densities of samples. When the mass ratio of POE to PP was 4/6, the apparent density of sample decreased by 6.7%. On one hand, the density of POE was smaller than PP. On the other hand, the addition of POE improved the melt strength of composites, which prevented the gas used for bubble nucleation and bubble growth from escaping from molten materials. Besides, the addition of microcrystalline wax also decreased the apparent densities of samples, which was similar with POE. However, with the increase of HDPE, the density of samples increased after an initial decrease and the minimum density was obtained at the mass ratio of 1/9, which was mainly due to the low melt strength leading to the loss of gas.

According to the data in Figure 10, the Vicat softening temperature of sample without polymer blends was 153.4 °C. But the Vicat softening temperature of sample decreased with the increase of the content of POE, HDPE or microcrystalline wax. On one hand, the Vicat softening temperatures of POE, HDPE and microcrystalline wax were lower than PP. On the other hand, the addition of POE, HDPE and microcrystalline wax decreased the crystallinity of PP, which also resulted in the decrease of the Vicat softening temperatures. When the mass ratio of HDPE to PP was 4/6, the Vicat softening temperature of the sample reached the minimum value 133.7 °C, which was 12.8% lower than that before blending.

## 4. Conclusions

In this study, the extrusion foaming process of PP/wood flour composites was studied. And the effects of the addition of POE, HDPE and microcrystalline wax on the rheological properties, cell morphology and mechanical properties of samples were investigated. The conclusions from this research are as follows:

1. The addition of HDPE and POE resulted in the two well-separated melting peaks in the DSC curves. But only POE could significantly increase both the oscillating shear modulus and the complex viscosity of the composites.
2. Compared with HDPE and microcrystalline wax, the sample with the introduction of POE could achieve the minimum cell size 78 μm and the maximum cell density 2.3 × 10^4^ cells/cm^3^, which caused a significant decrease of 58% in the cell size and a great increase of 170% in the cell population density. But the velocity difference of composites flowing in the sheet die made the shape of bubbles irregular. The bubbles on the radial section were stretched into the shape similar with the convex lens. Besides, the bubbles gradually became crescent-shaped along the extrusion direction.
3. Compared with the tensile and flexural property, the impact strength of the samples was significantly improved with the addition of POE. And the impact strength of sample blended with POE at the ratio of 6/4 increased by 84% compared with the sample without blending. Besides, the addition of microcrystalline wax contributed to the minimum apparent density of sample with the decline of 9%. Meanwhile, among the three polymer blends, the addition of HDPE contributed to the minimum Vicat softening temperature 133.7 °C at the blending ratio of 6/4.

However, compared with the samples prepared by the batching foaming process [31,32,33], the foamed wood-plastic samples prepared in this article have not reached the standard of microporous plastics in terms of cell size and cell density. Therefore, the future research needs to focus on the effects of the nucleating agent, interface modification and other aspects, so as to realize the preparation of microporous wood-plastic products with large cross-section. And the wood-plastic products with micro-porous structure can meet the requirements of high strength and light weight components in automotive, high-speed rail, aerospace, military and other fields by virtue of their excellent cost-effective ratio and high specific strength.

## Figures and Tables

**Table 1:** Compositions of the polymer blends.

Sample	Weight (%)			
	PP	POE	HDPE	Microcrystalline Wax
1	100	-	-	-
2	90	10	-	-
3	80	20	-	-
4	70	30	-	-
5	60	40	-	-
6	90	-	10	-
7	80	-	20	-
8	70	-	30	-
9	60	-	40	-
10	99	-	-	1
11	98	-	-	2
12	97	-	-	3

## Nomenclature

PP	Polypropylene	$∆H_{f}$	Heat of fusion generated by the cold crystallization
WF	Wood flour	$∆H_{f}^{o}$	Theoretical heat of fusion of 100% crystalline PP
POE	Polyolefin elastomer	ω	Weight fraction of PP in the sample
HDPE	High-density polyethylene	G′	Shear storage modulus
DSC	Differential Scanning Calorimeter	G″	Loss modulus
PVC	Polyvinyl chloride	η	Complex viscosity
PS	Polystyrene	$\bar{d}$	Number average diameter of all cells in one micrograph
PBT	Polybutylene terephthalate	d_i_	Diameter of a single cell
PTFE	Polytetrafluoroethylene	n	Number of counted cells
MAH-g-PP	Maleic anhydride-g-PP	N_0_	Number of cells per unit volume of the polymer
AC	Azodicarbonamide	A	Area of an SEM graph
WPC	Wood-polymer composites	M	Magnification factor
T_m_	Melting temperature	$\rho_{0}$	Density of the unfoamed sample
T_c_	Crystallization temperature	$\rho_{f}$	Density of the foamed sample
X_c_	Crystallinity percentage	HDT	Hot Deformation Temperature
